# Boolean network-based model of the Bcl-2 family mediated MOMP regulation

**DOI:** 10.1186/1742-4682-10-40

**Published:** 2013-06-14

**Authors:** Tomas Tokar, Zdenko Turcan, Jozef Ulicny

**Affiliations:** 1 Department of Biophysics, University of PJ Safarik, Jesenna 5, 040 01, Kosice, Slovakia

**Keywords:** Boolean network, Bcl-2 family, Apoptosis, Mitochondrial outer membrane permeabilisation

## Abstract

**Background:**

Mitochondrial outer membrane permeabilization (MOMP) is one of the most important points in the majority of apoptotic signaling cascades and it is controlled by a network of interactions between the members of the Bcl-2 family.

**Methods:**

To understand the role of individual members of this family within the MOMP regulation, we have constructed a Boolean network-based model of interactions between the Bcl-2 proteins.

**Results:**

Computational simulations have revealed the existence of trapping states which, independently from the incoming stimuli, block the occurrence of MOMP. Our results emphasize the role of the antiapoptotic protein Mcl-1 in the majority of these configurations. We demonstrate here the importance of the Bid and Bim for activation of effectors Bax and Bak, and the irreversibility of this activation. The model further points to the antiapoptotic protein Bcl-w as a key factor preventing Bax activation.

**Conclusions:**

In spite of relative simplicity, the Boolean network-based model provides useful insight into main functioning logic of the Bcl-2 switch, consistent with experimental findings.

## Introduction

Apoptosis is a process of programmed cellular death, distinct from necrosis [1,2], which can be well distinguished by its morphology [3]. It is an important homeostatic mechanism, and its defects may cause a variety of serious diseases, including neurodegenerative disorders [4], autoimmune diseases [5], or even cancer [6-8]. Signals leading to an apoptosis initiation can originate from an extracellular environment or from a cell’s internal space [8,9]. Apoptotic signals further proceed through an apoptotic signaling and regulatory network, that contains several control points [8,9]. One, highly important of such points is formed by a family of Bcl-2 (B-cell lymphoma 2) proteins [10,11]. An interplay between the Bcl-2 family’s members controls one of the most crucial apoptotic events - the mitochondrial outer membrane permeabilization (MOMP) [12,13].

MOMP allows the release of apoptotic key players - Smac/DIABLO and a cytochrome c, from the mitochondrial, intermembrane space to the cytosol [12,13]. In the presence of ATP, released cytochrome c binds to a cytosolic protein Apaf-1, causing Apaf-1 oligomerization and the recruitment of an inactive pro-caspase-9, leading to the formation of a multi-protein complex known as an apoptosome [14-16]. Within the apoptosome, pro-caspase-9 subsequently undergoes processing and activation [14-16]. The active caspase-9 proteolytically activates caspase-3 [17]. Smac/DIABLO, once released to the cytosol, inhibits XIAP (X-linked inhibitor of apoptosis) - the most prominent suppressor of caspases -3 and -9 [18]. Caspase-3 and other effector caspases (caspases -6 and -7) are the primary executioners of apoptosis [8,19]. Activation of these makes the point of no-return, after which the irreversible phase of apoptosis is executed [20]. Although other, mitochondria-independent apoptotic signaling pathways also exist [21], the mitochondrial (also known as intrinsic) pathway is the major one [22].

The MOMP is carried in “all or nothing” manner, where no intermediate MOMP states are possible. A control mechanism of such an event can be modeled by, in terms of complex systems science, a bistable switch.

This interesting property has made the Bcl-2 family an attractive subject of mathematical modeling and computer simulations. There are several works regarding modeling and a simulation of the Bcl-2 family and the control of MOMP, revealing and examining a variety of non-linear system behaviors such as robustness, stimulus-response ultrasensitivity [23] and bistability [24-26]. Besides these, the Bcl-2 family was involved in several other, more general models of apoptosis signaling [27-29].

All the above-mentioned models are continuous, dynamically simulating the chemical reaction kinetics of the studied system. These models reduce their complexity through aggregating proteins with similar function into functional groups. The most prominent group member is taken as the representative of the given group. Although the above-mentioned models of Bcl-2 regulatory network are of various levels of detail, they all adopt such simplification. This is done usually by grouping the Bcl-2 family’s members into three or four groups according to their structural and functional classification. Such division provides reasonable trade-offs between the model’s simplicity and plausibility. However, when grouped together, certain important functional specifics are ignored.

The critical factor which limits the development of more detailed, quantitative models of the Bcl-2 family is the availability of quantitative data, that is still a systems biology bottleneck [30]. However, the works of Chen et al [31] and Dai et al [32] provided affinity measurements of most Bcl-2 protein–protein bindings – major type of Bcl-2 intra-familiar interactions. Furthermore, Dussmann and colleagues [33] measured single-cell dynamics of MOMP commitment and supported his measurements by Bcl-2 family model similar to those mentioned above. Recently, Lindner et al [34] translated the western-blot quantifications of several Bcl-2 proteins and clinical findings into currently the most detailed model of great predictive power.

Despite the ever growing amount of experimental data, there remain quantitative parameters that need to be supplemented by relevant experimental *in vivo* measurements. The absence of these parameters provides an opportunity for less demanding qualitative description using the discrete state models. In this work we propose such model of the Bcl-2 family mediated regulation of MOMP based on Boolean network modeling. The Boolean network (BN) approach is one of the best suited approaches to the qualitative modeling of complex biological systems [30,35]. BN, first introduced in the late 1960s [36], was originally used to model gene regulatory networks and signaling pathways [37]. Although, BN does not model continuous time dynamics of the studied system, it may reveal properties of state transition dynamics [37]. For the first time BN model involving members of the Bcl-2 family appeared in work of Calzolari et al [38]. Mai and Liu [35] and few months later Schlatter et al [30], published the most recent BN-based models of apoptosis, both containing simplified mechanism of Bcl-2 family MOMP control. However, as far as we know, no comprehensive modeling work involving the whole Bcl-2 family has been published yet.

## Modeling and simulations

### Model and its biological relevance

Bcl-2 family’s members are functionally classified as either antiapoptotic, or proapoptotic. Structurally, Bcl-2 proteins can be categorized according to the number of Bcl-2 homology domains (BH) in their *α*-helical regions [8,39]. Antiapoptotic members (Mcl-1, A1, Bcl-xL, Bcl-2, Bcl-w and Bcl-B) are characterized by the presence of four BH domains (BH1-4) [40,41]. Their role is to prevent MOMP by inhibition of proapoptotic family members [40,41]. Proapoptotic members can be divided to BH3-only proteins and multidomain proteins - effectors [8]. BH3-only proteins can be further subdivided based upon their role in apoptotic signaling. BH3-only subgroup members, termed sensitizers (Noxa, Bad, Puma, Hrk, Bmf and Bik), can only bind to antiapoptotic Bcl-2 proteins, forming inactive dimers [39]. Members of another BH3 subgroup, termed activators (Bim and Bid), can act in the same way [39], but in addition, activators can directly activate effectors [40,42]. Effectors, once activated, undergo oligomerization and form pores in mitochondrial outer membrane (MOM), leading eventually to MOMP. [13,43]. Therefore, effectors are the primary target of inhibition by their antiapoptotic relatives [42].

Altogether, interactions between Bcl-2 family members can be classified into only three types: i) Binding and mutual inhibition between antiapoptotic and BH3-only proteins. ii) Binding and mutual inhibition between antiapoptotic proteins and effectors. iii) Activation of effectors by BH3-only proteins. However, the situation ceases be so simple when we focus on the interaction between individual molecules. E.g., the BH3-only sensitizer Noxa can bind to and inhibit only two antiapoptotic proteins (see Table 1) [8,31,39], but the other BH3-only sensitizer, Puma is able to inhibit five of six major antiapoptotic proteins [8,31,39]. On the other hand, while it seems that the antiapoptotic protein Bcl-B is not bound or inhibited by any of the BH3-only proteins [44], the other antiapoptotic protein Bcl-xL is bound by seven of them [8,31]. There is also a strong asymmetry in the level of inhibition of effectors by antiapoptotic proteins. While Bak is inhibited only by three antiapoptotic proteins, Bax is inhibited by all six of them [8,39].

**Table 1:** Binding and inhibition between individual members of the Bcl-2 family

**Bcl-2**	**Full name of the protein**	**Binds to and inhibits**	**Ref.**
**protein**			
*Antiapoptotic*			
*Members:*			
Mcl-1	Myeloid cell leukemia sequence-1	Noxa, Bim, Puma, Bax, Bak	[8,31]
Bcl-2	B-cell lymphoma 2	Bad, Bim, Puma, Bmf, Bax	[8,31]
A1	Bcl-2 related protein	Noxa, Bim, Puma, tBid, Hrk, Bik, Bax, Bak	[8,31]
Bcl-xL	Bcl-2-like	Bad, Bim, Puma, tBid, Hrk, Bmf, Bik, Bak, Bax	[8,31]
Bcl-w	Bcl-2-like-2	Bad, Bim, Puma, tBid, Hrk, Bmf, Bik, Bax	[8,31]
Bcl-B	Bcl-2-like-10	Bax	[44]
*BH3-only*			
*Members:*			
Noxa	Phorbol-12-myristate-13-acetate-induced	Mcl-1, A1	[8,31,39]
	protein 1		
Bad	Bcl-2 antagonist of cell death	Bcl-xL, Bcl-w, Bcl-2	[8,31,39]
Bim	Bcl-2like-11	Bcl-xL, Bcl-w, Bcl-2, Mcl-1, A1	[8][31,39]
Puma	Bcl-2-binding component-3	Bcl-xL, Bcl-w, Bcl-2, Mcl-1, A1	[8,31,39]
tBid	truncated BH3-interacting	Bcl-xL, Bcl-w, A1	[31,39]
	domain death agonist		
Hrk	Harakiri	Bcl-xL, Bcl-w, A1	[31]
Bmf	Bcl-2-modifying factor	Bcl-xL, Bcl-w, Bcl-2	[31,39]
Bik	Bcl-2-interacting killer	Bcl-xL, Bcl-w, A1	[31]
*Effectors:*			
Bak	Bcl-2-antagonist/killer-1	Bcl-xL, Mcl-1, A1	[8,39]
Bax	Bcl-2-associated X protein	Bcl-xL, Bcl-w, Bcl-2, Bcl-B, Mcl-1, A1	[8,39]

The knowledge about interactions between Bcl-2 family members was encoded in the Boolean network-based model we present here. The model contains 14 nodes, representing the Bcl-2 family’s members. Each member of the Bcl-2 family is represented by one of the nodes. The only exception was Bad & Bmf and Hrk & Bik pairs, coupled together due to their identical intra-familiar interaction profiles (see Table 1).

The whole model contains 34 connections between nodes, each representing one molecular interaction.

### Transition rules

Each of the nodes can be either active or inactive. Each of the nodes is affected by received inputs from one or several other upstream nodes. The state of the node *i* in the next time step *s*_*i*_(*t*+1) is defined by the following transition rule:

(1) $$\begin{array}{ll} s_{i}(t+1) & =\left\{\begin{array}{ll} 1, & \Delta_{i}>0,\\ s_{i}(t), & \Delta_{i}=0,\\ 0, & \Delta_{i}<0, \end{array}\right)\; \end{array}$$

(2) $$\Delta_{i}=e_{i}+\underset{j}{\sum }r_{\text{ij}}\;s_{j}(t)$$

**Figure 1:**
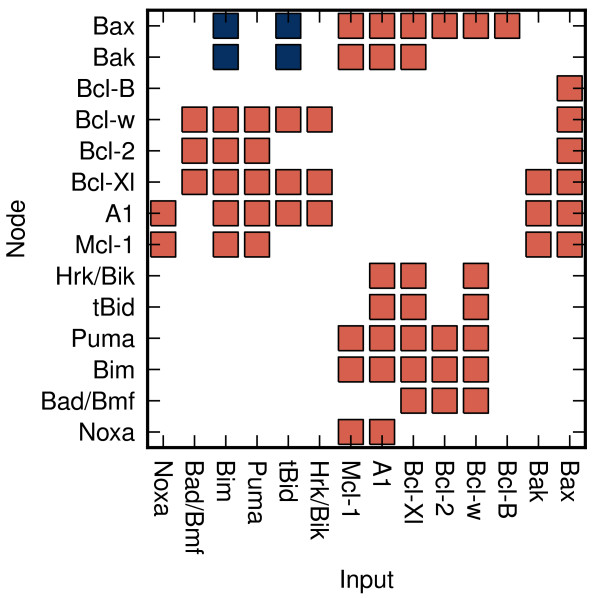
**Relationships between the nodes of the model.** The red squares represent the negative relationships of the inputs toward the related nodes. Negative relationship corresponds to mutual binding and inhibition between two members of the Bcl-2 family (see Table 1). The blue squares represent the positive relationships of the inputs toward the related nodes. Positive relation corresponds to activation of the effectors Bax/Bak by certain BH3-only proteins - activators (tBid, Bim) [40,41].

Here, *r*_*ij*_ specifies the relation of the *j*-th node to *i*-th node, and it may have three possible values: *r*_*ij*_=1 if *j*-th node activates *i*-th node, *r*_*ij*_=−1 if *j*-th node inhibits *i*-th node and *r*_*ij*_=0, if nodes *j* and *i* are not connected (the relationships between nodes are depicted in the Figure 1). The value of *e*_*i*_ defines the expression of the protein represented by the *i*-th node (see following section).

Since Bcl-2 family members inhibit each other by mutual binding and formation of inactive dimers, our model treats the inhibitory relationships between two nodes as bipartite (if *r*_*ij*_=−1, then *r*_*ji*_=−1).

During the simulation, the states of all nodes are step-wise simultaneously reevaluated according to the transition rule described by the eq. (1), until the simulation is terminated.

### Influence of the external conditions

The transition function of the *i*-th node is dependent on the value of *e*_*i*_. The value of binary vector *E* ($E=\{e_{1},e_{2},\ldots e_{16}\}$) represents here, what we termed the “expression” of the Bcl-2 family proteins. The value *e*_*i*_=1, corresponds to the cellular conditions allowing the synthesis and, if required, the post-translational/post-transcriptional activation (e. g. activation of Bid requires proteolytic cleavage by Caspase-8 [45]) of the *i*-th protein. Alternatively, the value *e*_*i*_=0, corresponds to the conditions preventing the synthesis and/or post-translations activation of the *i*-th protein.

Since the model contains 14 nodes, the vector of expressions can have 2^14^=16384 possible values. However, since other than BH3-only mediated activation of Bax/Bak is irrelevant to our work (the subject of our study is the Bcl-2 family mediated regulation of MOMP), we exclude here the expression vectors where *e*_*Bax*_=1 or *e*_*Bak*_=1, reducing thus the number of possible values to 2^12^=4096. The value of the vector *E* remains constant during each simulation.

### Terminal states

The simulation is terminated at the time *t* once the state of the model *S* ($S=\{s_{1},s_{2},\ldots s_{14}\}$) satisfies the following condition:

(3) S(t)=S(t−n),n=1,2,…

The condition described in the eq. (3), can imply either that the model converged to the steady-state (*n*=1), the model is oscillating between two different states (*n*=2), or the model is periodically orbiting through the set of states (*n*>2).

The state *S*(*t*), satisfying the condition (3) is the model’s terminal state *S*(*t*_*end*_). If the *S*(*t*_*end*_) involves the states of both effectors, *s*_*Bax*_(*t*_*end*_)=0 and *s*_*Bak*_(*t*_*end*_)=0, then the *S*(*t*_*end*_) is denoted as the “survival” state. If the *S*(*t*_*end*_) involves the states of one of the effectors, either *s*_*Bax*_(*t*_*end*_)=1, or *s*_*Bak*_(*t*_*end*_)=1, then the *S*(*t*_*end*_) is simply denoted as the “pro-MOMP” state.

## Results

### We have identified 1046 of the “survival” states, in which the model preserve the effectors Bak and Bax inactive

The very first step was to find the terminal states in which the model is allowed to persist without the activation of effectors (Bak and Bax) - survival states.

Therefore, for each of the 4096 expression vectors we performed 4096 simulations, each simulation starting from one of the 4096 of the initial states (4096=2^12^, that is the number of possible initial states, including both *s*_*Bax*_(*t*_0_)=0 and *s*_*Bak*_(*t*_0_)=0).

We have identified 1046 unique survival states. The 388 of these states are logical steady-states, remaining 678 of the survival states are oscillating. Hereafter, we assume that these 1046 states represent basal, cellular conditions. To lead the cell out from such survival state it requires the change of an expression vector which would initiate the state transition. In the next step, we have investigated the transitions from the survival states to other terminal states. To analyze these transitions, for each of the 1046 survival states, we performed 4096 simulations. In each simulation we used one of the 4096 expression vectors and one of the survival states to define the initial conditions. Thus we “exposed” individual survival states to all the expression vectors and simulated the effect of changes of cellular conditions on actual state of the Bcl-2 proteins family.

Around 70% of the 4.2 million (1046×4096) simulations led to survival, the remaining 30% of the simulations led to pro-MOMP terminal states, i.e. the states where at least one of the effectors was found active.

### We found 200 of the trapping states

During the analysis of the transitions between the survival states and the pro-MOMP states, we have revealed an interesting finding. We have discovered the existence of 200 survival states from which the model cannot achieve the pro-MOMP states, regardless of the vector of expressions. Moreover, the model, once found in such a “trapping” state, can only be transitioned to another trapping state. The trapping of the Bcl-2 regulatory mechanism in one of these states would cause fatal malfunctioning of the MOMP regulation. The cell arrested in one of such states becomes resistant to external apoptotic stimuli - a condition which is one of the hallmarks of cancer cells [46]. Therefore, we denoted these states as the “tumor” states.

**Figure 2:**
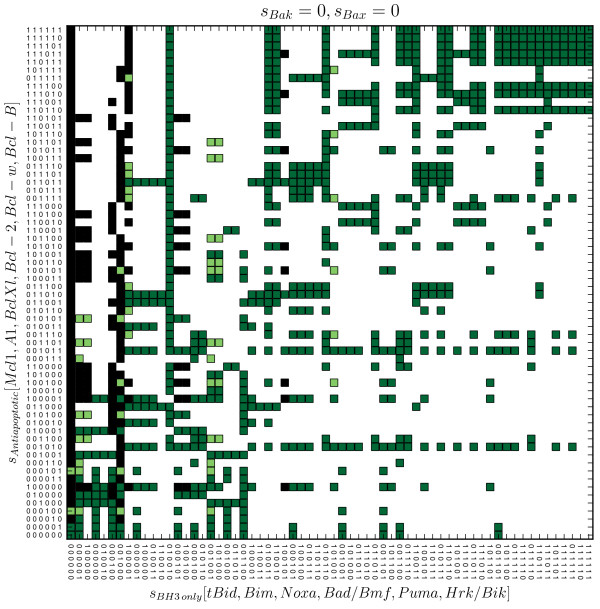
**Model’s survival states.** The figure depicts the survival states – Bax, Bak representing nodes are inactive. The squares are depicting the terminal states of the model within the “configuration space”, where the configurations of states of nodes representing the antiapoptotic and BH3-only proteins are arranged along y- and x-axis, respectively. The black squares represent the “tumor” states, while the green squares represent the functionally “semioptimal” (light green) and “optimal” (dark green) survival states. While the first mentioned allow only the activation of Bak (transition *T*_4_ in Figure 3), but not Bax, “optimal” states allow activation of both effectors.

In general, these states (the black squares in the Figure 2) exhibit strong imbalance in activity of antiapoptotic vs BH3-only proteins. BH3-only proteins are overwhelmed by activity of their antiapoptotic relatives. The activity of these compensates the positive influence of the expression vectors on BH3-only proteins. According to the rule described in eq. 1, in such a case the inactive BH3-only proteins remain inactive regardless of the configuration of expression vectors. Therefore, changing the expression vector results only in relocation of the model from one tumor state to another state, which will be similarly imbalanced.

**Figure 3:**
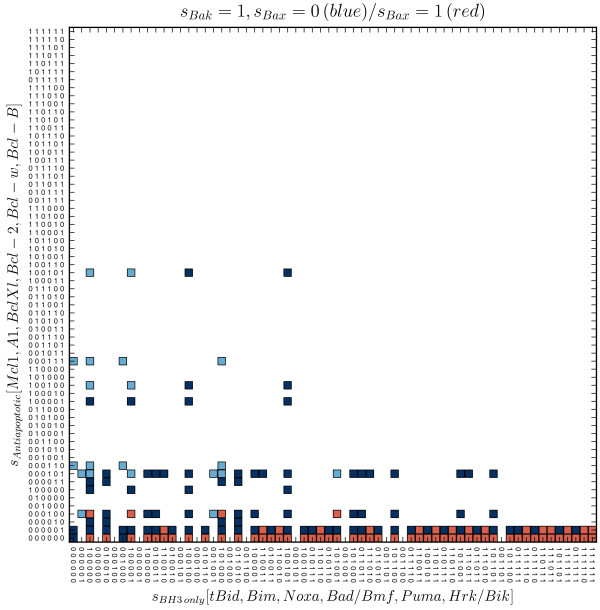
**Model’s non-survival states.** The figure depicts the non-survival states – either the Bax, or Bak is active (blue squares), or both of them are active (red squares). The dark blue squares represent those states, which allow subsequent activation of Bax, initiated by the change in the expressions. In contrast, the states represented by the light blue squares suffer from the inability to allow the activation of Bax.

As we found, the cell can be “liberated” from a trapping state by pharmacological inhibition of activity of antiapoptotic proteins (e.g. by competitive inhibition to prevent neutralization of proapoptotic proteins). Especially effective would be the inhibition of the Mcl-1, since the tumor states are overly abundant among the survival states involving the activity of Mcl-1.

The existence of trapping tumor states indicates that relationships in Bcl-2 family allow the establishment of the molecular populations of the Bcl-2 proteins which could be very insensitive to apoptotic signaling. The apparent relationship between the activity of the Mcl-1 and tumor states suggests that inhibition of Mcl-1 can be of special therapeutic relevance of targeting tumor cells.

### There are two functionally distinct subsets of survival states. Those which allow model to activate the Bak, but not Bax and the states allowing activation of both effectors

The remaining 846 survival states can further be classified in two groups. The first group consists of 54 survival states, from which the model can relocate to pro-MOMP states where the only active effector is Bak. From the 792 survival states of the second group, the model can be turned to states with a single effector (Bak) activity, as well as to the pro-MOMP states, where both effectors are active. The first group we denote as “semioptimal” (the light green squares in Figure 2), the second one we denote as “optimal” (the dark green squares in Figure 2).

Similarly, we distinguish several functionally distinct subsets among the pro-MOMP states. Firstly, every pro-MOMP state may be classified according to the activity of effectors. We have found 108 of the pro-MOMP states in which Bak is active, but Bax remains inactive (the blue squares in Figure 3). Besides these, we also have found 132 of the pro-MOMP states, in which both effectors are active (the red squares in Figure 3). However, we haven’t found any such terminal state, where the Bax was active, while the Bak not, indicating that such state is unaccessible by the model, regardless of expressions or initial conditions.

Secondly, the first of the mentioned groups – Bak-active only, can further be divided in two functionally distinct subgroups: states which allow additional activation of Bax (the light blue squares in Figure 3), and those which don’t (the dark blue squares in Figure 3).

It is very interesting that, while the first subgroup is accessible only from the “optimal” survival states, the second one, can be accessed from both, “optimal” and “semioptimal” survival states.

### Survival to MOMP transition is irreversible

We have performed another series of simulations in which each of the pro-MOMP states was used as the initial state of the model and each of the 4096 expression vectors were iteratively applied to the model. We have found that it is impossible to turn the model from any of the pro-MOMP states back to the survival one, regardless of the expression vectors.

Irreversibility of the transition to pro-MOMP states originates in activity of effectors itself. Mutual inhibitory relationships between effectors and corresponding antiapoptotic proteins compensate the influence of the expression vectors on a given antiapoptotic protein. According to the rule described in eq. 1, in such a case inactive antiapoptotic protein remains inactive and inhibition of effectors remains insufficient to suppress their activity, regardless of the expression vector.

### The transitions from the survival to MOMP are caused by expression changes of four distinct types

**Figure 4:**
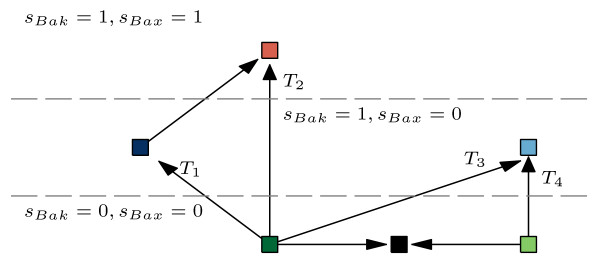
**Transitions between the individual subsets of model’s terminal states.** Using the same color notation as in the Figures 2 and 3, the types of the model’s transitions between five functionally distinct subgroups of model’s states are depicted.

When taking previous results together, we may distinguish six distinct groups of model’s terminal states. Three of these groups associate survival states, characterized by no effectors activity. Two groups associate pro-MOMP states, involving the activity of Bak, but not Bax. The sixth group associates the states involving the activity of both effectors. Assuming that the single effector activation is sufficient for the MOMP occurrence, four types of survival-to-MOMP transitions can be distinguished (*T*_1_ – *T*_4_, see Figure 4).

We have analyzed the influence of the expression of the given protein on the initiation of these transitions by means of multiple correlation coefficient - *R*^2^ (for more details see Appendix: A calculation of multiple determination coefficients).

**Figure 5:**
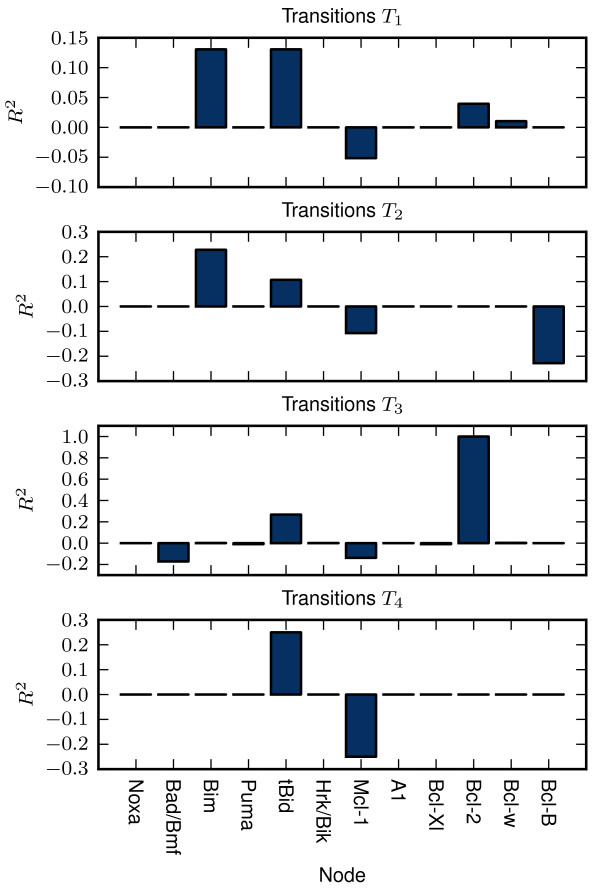
**Multiple determination coefficients.***R*^2^ of the protein expressions calculated across the sets of unique expressions vectors causing the survival-to-MOMP transitions of given type (for more details see Appendix: A calculation of multiple determination coefficients).

Figure 5 shows that regardless of the transition type, the necessary condition for the transition to MOMP is the expression of at least one of the activators (Bim, tBid). On the other hand it seems that the expression of the Mcl-1 is the most significant factor that prevents the MOMP. The statistical importance of the expression of activators and the absence of Mcl-1 is common for both *T*_1_ and *T*_2_ transitions. Nevertheless, the transitions of type *T*_2_ - the activation of both effectors, additionally requires the absence of Bcl-B expression. This finding is not unexpected as the Bcl-B is the only inhibitor of Bax that is not suppressed by any of the BH3-only proteins [44]. Furthermore, the model predicts that after the *T*_1_ transition, the subsequent downregulation of Bcl-B can cause the additional activation of Bax (the arrow pointing from the dark blue square to the red one, Figure 4).

Transitions of the type *T*_3_ are similar to the *T*_1_ and *T*_2_. The only difference is that transitions of the type *T*_3_ occurs only in presence of the Bcl-2 expression. In other words, expression of the antiapoptotic protein Bcl-2 prevents the transitions of type *T*_3_ and *T*_4_– transitions from optimal survival states to “dark blue” and “red” pro-MOMP states. If model is located in one of the “light blue” states, subsequent down-regulation of Bcl-2 would allow its relocation to “dark blue” states (not depicted in Figure 4), but not to “red” states.

The transitions of type *T*_4_ are caused by lack of the Mcl-1 expression while involving the tBid expression. Since the number of semioptimal states is very small, the probability of *T*_4_ transitions is low.

We have found (data not shown) that the trapping of the model within the “trapping” state occurs as the antiapoptotic expression disproportionately dominates over the expression of BH3-only proteins. This points to the necessity of the balance between the presence and synthesis of the both pro- and antiapoptotic Bcl-2 proteins within the cell.

## Discussion

We have analyzed the Bcl-2 family interaction network using the Boolean network-based computational model. Bcl-2 family members have been represented by nodes, with binary encoded activity. The active (ON state) of the given node represents the biologically active form of the represented protein. Nodes are mutually interacting according to the given transition rules and the pre-defined relationship matrix, representing the currently known interactions among the Bcl-2 proteins. In addition, the model operates under the influence of the vector of expressions that represent the biological conditions. Change of vector of expressions is the primary driving force of the Bcl-2 regulatory mechanism. The expression vector allows introduction of synthesis and the post-translational/-transcriptional activation of zymogens, if relevant for given protein.

Computational simulations of the model show that the majority of the expression vectors lead the Bcl-2 family into set of states avoiding Bax and/or Bak activation. Our results suggest, that once the antiapoptotic proteins significantly outnumber the BH3-only proteins’ activity, the Bcl-2 family regulation may be seriously disrupted. Our model predicts, that once this happens, even the subsequent activation of proapoptotic BH3-only proteins cannot recover the proper MOMP. We have noted that the defects of MOMP regulation are often associated with presence of Mcl-1 activity. However, the existence of such “tumor facilitating” trap shows the importance of the balance between pro- and antiapoptotic proteins maintained by their continuous expression.

Depending on the current state of the Bcl-2 family, certain configurations of the incoming signals cause the activation of effectors Bak, and/or Bax. Statistically, the most important signals are the truncation of Bid to tBid, the activation of Bim and the downregulation of Mcl-1.

The ability of tBid to initiate apoptosis through MOMP has been well documented by numerous works [45,47]. Similarly, extensive experimental support exists, proving that activation of Bim [48-50] and downregulation of Mcl-1 [51,52] lead to apoptosis in cells.

It seems that the BH3-only mediated activation of Bak requires less specific conditions, compared to the activation of Bax. This finding is well supported by several experimental works, suggesting that MOMP initiated by BH3-only proteins occurs mainly through the activation of Bak, not Bax [53-56]. According to the model, the activation of Bax is associated with the downregulation of Bcl-w. Finally, our results confirm the irreversibility of the effectors activation, which has been previously experimentally shown [57-59].

In spite of the simplicity of the Boolean-based approach the model provides remarkable predictive and explanatory power. Moreover, the proposed model can be reutilized for further analyses of robustness and stability of Bcl-2 family regulation of apoptosis.

## Appendix: A calculation of multiple determination coefficients

This analysis is utilized to compare the importance of the expression of particular proteins, with respect to the transition from certain group of states to another group of states.

Let’s have set of *n* unique expression vectors – $E=\{e_{1},e_{2},\ldots e_{12}\}$ that cause the studied transition. For each couple of nodes *i*, *j* we can calculate the phi coefficient:

(4) $$\phi_{\text{ij}}=\frac{n_{11}n_{00}-n_{10}n_{01}}{\sqrt{n_{1∙}n_{0∙}n_{∙0}n_{∙1}}},$$

where *n*_00_, *n*_01_, *n*_10_, *n*_11_, are counts of the following combinations of values *e*_*i*_, *e*_*j*_ across the set of expression vectors:

$$\begin{array}{cccc} & e_{i}=1 & e_{i}=0 & \text{total}\\ e_{j}=1 & n_{11} & n_{10} & n_{1∙}\\ e_{j}=0 & n_{01} & n_{00} & n_{0∙}\\ \text{total} & n_{∙1} & n_{∙0} & n \end{array}$$

The phi coefficient is a measure of association for two binary variables, similar to Pearson correlation coefficient [60].

The matrix of phi coefficients - *R*_*ϕ*_, is then used as to calculate the coefficient of multiple determination – *R*^2^:

(5) $$R_{i}^{2}=c_{i}^{T}R_{\phi ,i}^{-1}\;c_{i},$$

where the *c*_*i*_ is the vector of values *ϕ*_*ij*_, *j*=1,2…12, *i*≠*j*. *c*_*i*_ is actually the vector of correlations between the independent variables and the target variable – *e*_*i*_. $c_{i}^{T}$ is the transpose of *c*. The *R*_*ϕ*,*i*_ is the matrix *R*_*ϕ*_, reduced by removing the *i*-th line and *i*-th column. *R*_*ϕ*,*i*_ is actually the matrix of correlations between the independent variables and $R_{\phi ,i}^{-1}$ is the inverse of the matrix *R*_*ϕ*,*i*_.

Finally, the $R_{i}^{2}$ was multiplied by −1 if the count of *e*_*i*_=0 appearances was greater than the count of *e*_*i*_=1, across the set of expressions – the expression of the *i*-th node was mostly absent among the expression vectors causing the given transition.

In case the values of *i*−*th* protein expression had no variability – either *e*_*i*_=0, or *e*_*i*_=1 among all the expression vectors, the $R_{i}^{2}$ was arbitrary set either to −1, or 1, respectively. The correlations of any of the other expression with the *e*_*i*_ were then excluded from any other calculations. Such situation occurs in case of expression of Bcl-2 among the transitions of the type *T*_3_ (see Figure 4).

## Competing interests

The authors declare that they have no competing interests.

## Authors’ contributions

TT proposed the Boolean networks-based model of Bcl-2 family, implemented the model within the Python environment and performed most of the simulations. Furthermore, TT processed and analyzed the obtained data, prepared all the illustrations and major part of the manuscript. ZT participated on the implementation of the model and its simulations. Furthermore, ZT contributed to this study by critical revision of the manuscript. JU substantively contributed to this work, by extensive revision of the manuscript. Furthermore, JU gave the final approval of the version to be published. In addition, all the authors have contributed to this work, by numerous valuable ideas and proposals. All authors read and approved the final manuscript.
